# A Structural View of SARS-CoV-2 RNA Replication Machinery: RNA Synthesis, Proofreading and Final Capping

**DOI:** 10.3390/cells9051267

**Published:** 2020-05-20

**Authors:** Maria Romano, Alessia Ruggiero, Flavia Squeglia, Giovanni Maga, Rita Berisio

**Affiliations:** 1 Institute of Biostructures and Bioimaging, IBB, CNR, 80134 Naples, Italy; maria.romano@cnr.it (M.R.); alessia.ruggiero@cnr.it (A.R.); flavia.squeglia@cnr.it (F.S.); 2 Institute of Molecular Genetics, IGM, CNR, 27100 Pavia, Italy; giovanni.maga@igm.cnr.it

**Keywords:** SARS-CoV-2, COVID19, RNA replication, protein structure, infectious disease

## Abstract

The current coronavirus disease-2019 (COVID-19) pandemic is due to the novel coronavirus SARS-CoV-2. The scientific community has mounted a strong response by accelerating research and innovation, and has quickly set the foundation for understanding the molecular determinants of the disease for the development of targeted therapeutic interventions. The replication of the viral genome within the infected cells is a key stage of the SARS-CoV-2 life cycle. It is a complex process involving the action of several viral and host proteins in order to perform RNA polymerization, proofreading and final capping. This review provides an update of the structural and functional data on the key actors of the replicatory machinery of SARS-CoV-2, to fill the gaps in the currently available structural data, which is mainly obtained through homology modeling. Moreover, learning from similar viruses, we collect data from the literature to reconstruct the pattern of interactions among the protein actors of the SARS-CoV-2 RNA polymerase machinery. Here, an important role is played by co-factors such as Nsp8 and Nsp10, not only as allosteric activators but also as molecular connectors that hold the entire machinery together to enhance the efficiency of RNA replication.

## 1. Introduction

Coronavirus disease-2019 (COVID-19) is a respiratory disease caused by a novel enveloped, positive-sense, single-stranded RNA betacoronavirus, denoted as SARS-CoV-2. In December 2019, a cluster of patients in the Chinese city of Wuhan was diagnosed with a pneumonia of unknown etiology. At the time of writing, SARS-CoV-2, has caused over 4 × 10^6^ confirmed cases and 2.98 × 10^5^ fatalities worldwide. The efficiency of disease transmission, the fact that a significant proportion of infected people develop pneumonia and the increased risk of lethality in fragile patients such as the elderly, patients with immunodeficiency and people affected by chronic respiratory and heart diseases, make SARS-CoV-2 infection a serious global health threat. Consequently, on January 2020, the World Health Organization (WHO) declared the situation a public health emergency of international concern and in March 2020, it declared COVID-19 a pandemic threat. The scientific community has responded promptly to the emergency by focusing heavily on accelerating research and innovation, as witnessed by the copious amount of recent literature. This has set the foundation for understanding the molecular determinants of the disease and the development of targeted therapeutic interventions [1,2,3]. These ground-breaking studies have shown that SARS-CoV-2 shares 79.5% of its genome with SARS-CoV [1], thus it is sufficiently divergent from SARS-CoV to be considered a new human-infecting betacoronavirus [3]. Genome sequence analysis has revealed SARS-CoV-2 phylogenetic relationships with bat-derived SARS-like coronaviruses, which suggests a zoonotic origin [1]. However, much of what we can infer about the biology of SARS-CoV-2 comes from previous studies on the SARS-CoV. Starting from these data, the molecular mechanisms underlying the evolution, adaptation, and spread of this virus warrant urgent investigation.

SARS-CoV-2 gets into the cell through recognition by the spike glycoprotein present on the surface of the virus envelope of the angiotensin converting enzyme 2 (ACE2) receptors, as previously observed for SARS-CoV [4,5]. It is possible that other receptors mediate the entry of SARS-CoV-2 into host cells, such as CD147 [6]. After attachment, the human transmembrane protease serine 2 (TMPRSS2) cleaves and activates the spike protein [7] in an event that allows SARS-CoV-2 to enter the cells by endocytosis or direct fusion of the viral envelope with the host membrane [8,9].

Once inside the cell, the infecting RNA acts as a messenger RNA (mRNA), which is then translated by host ribosomes to produce the viral replicative enzymes, which generate new RNA genomes and the mRNAs for the synthesis of the components necessary to assemble the new viral particles. SARS-CoV-2 replication is a complex process that involves RNA synthesis, proofreading and capping. Similar to other viruses, this process is likely to actively involve many host proteins, like DDX helicases, which are exploited by the virus for more efficient replication [10,11,12]. Understanding the molecular mechanisms that guide the replication of this coronavirus is essential in order to develop therapeutic tools to neutralize SARS-CoV-2. Here, we review structural information, mostly obtained through homology modeling based on the available structures for other coronaviruses, on the main protein actors of SARS-CoV-2 RNA replication and transcription.

## 2. Organization of SARS-CoV-2 Genome

Like other coronaviruses, SARS-CoV-2 has a positive-sense single-stranded genomic RNA, approximately 30 kb in length [13], which is among the largest known RNA genomes.

**Figure 1:**
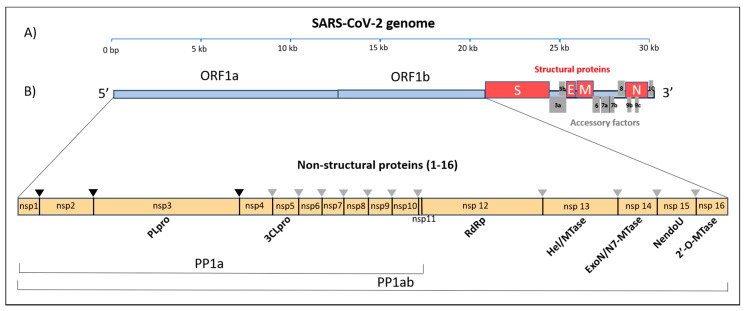
SARS-CoV-2 19 polycistronic genome. (**A**) Genome of SARS-COV-2 organized in individual ORFs. (**B**) Polyprotein 1ab (PP1ab) embeds 16 non-structural proteins (Nsps); the black and grey triangles indicate the cleavage sites of the protease PLpro and 3CLpro, respectively. Names of confirmed and putative functional domains in the Nsps are also indicated.

The genomic RNA (gRNA) has a 5′-cap and a 3′-poly(A) tail and can act as an mRNA for immediate translation of the viral polyproteins. In addition, both 5′- and 3′-ends of the gRNA present a highly structured untranslated region (UTR) that plays an important role in the regulation of RNA replication and transcription. Seven stem-loop structures are present at the 5′-UTR, while a stem-loop and a pseudoknot are present at the 3′-UTR. These two latter structures are mutually exclusive, since their sequences overlap. It is hypothesized that the alternate formation of either the pseudoknot or the stem-loop play some role in the transcriptional regulation [14]. The SARS-CoV-2 genome contains 14 open reading frames (ORFs), preceded by transcriptional regulatory sequences (TRSs). The two main transcriptional units, ORF1a and ORF1ab, encode replicase polyprotein 1a (PP1a) and polyprotein 1ab (PP1ab), respectively (Figure 1). The largest polyprotein PP1ab embeds non-structural proteins (Nsp1-16), which form the complex replicase machinery. This includes enzyme activities that are rare or absent in other families of positive-stranded (+) RNA viruses [15]. At the 3′ end, the viral genome encodes four structural proteins (spike, envelope, membrane, nucleocapsid), which are components of the mature virus and play a crucial role in viral structure integrity, or as in the case of the spike protein, for viral entry into the host [4,5,6]. Interspersed among the structural genes, the 3′ end of the genome also contains nine putative ORFs for accessory factors [16] (Figure 1). The structural and accessory proteins are translated from a set of nested sub-genomic (g) RNAs. all terminating with the 3′-end of the full-length gRNA. The generation of these sgRNAs starting from negative-sense RNA intermediates is regulated by the TRSs. During minus-strand RNA synthesis, the viral RNA polymerase pauses at each TRS sequence. The pause can be resolved either by continuing the synthesis through the TRS into the adjacent gene, or it can lead to the termination of transcription with the generation of a sgRNA. The exact molecular mechanisms that determine either outcome are yet to be fully clarified, but they likely involve long-range RNA-RNA interactions between complementary sequences [17,18].

## 3. A Structural View at SARS-CoV-2 RNA Replicatory Machinery

### 3.1. RNA Machinery as a Whole: Nsp Interaction Pattern

Coronavirus RNA synthesis is performed by the replication-transcription complex (RTC), associated with a complex vesicular network [14]. Such membranous structures comprise convoluted membranes (CVs) and double-membrane vesicles (DMVs), originating from the endoplasmic reticulum. These different membranous elements correlate with a precise spatial distribution of the different components of the RTC. The viral replication machinery is anchored to the CVs, thanks to the transmembrane proteins Nsp3, Nsp4 and Nsp6, while the dsRNA originating from the replication-transcription process is mainly contained within the DMVs. This suggests that these latter structures act as a protective environment to avoid detection of dsRNA by innate immunity sensors, and subsequent degradation [19].

The viral RNA replication machinery of SARS-CoV-2 involves an array of functional proteins from the N- to C-termini of the polyprotein, PP1ab (Figure 1). These include the essential RNA-dependent RNA polymerase (RdRp, Nsp12) [20], the zinc-binding helicase (HEL, Nsp13) [21] and a number of other enzymatic functions related to viral RNA modification, such as mRNA capping (Nsp14, Nsp16), RNA proofreading (Nsp14) [22,23,24], and uridylate-specific endoribonuclease activity (NendoU, Nsp15), which has been shown to counteract double strand RNA sensing [25,26,27]. The activity of these enzymes is further regulated by the association with other non-structural proteins (Nsp7–Nsp10) that are likely necessary to achieve all of the replication and transcription processes [28,29,30]. As observed for other nidoviruses, all of these protein subunits likely associate in a replication transcription enzyme complex anchored to membranes derived from the host cell ER [19,31], which drives the synthesis of new genome molecules and also sub-genomic (sg) messenger RNAs (mRNAs) [32]. Table 1 reports all structural information hitherto available for the main non-structural proteins involved in SARS-CoV-2 RNA replication and its homologs, together with their proposed functions.

In addition to these main RNA replication functions, other activities are important to enhance the efficiency of the whole machinery. Suppression of host gene expression and blockage of innate immune responses in infected cells have been attributed to Nsp1, which is considered a major CoV virulence factor [33,34]. Also, the primary role of the nucleocapsid N protein is to protect the viral genome by packing it into a helical ribonucleocapsid (RNP) [35,36]. Accordingly, the N protein must tightly bind the RNA, even though it is exposed during viral infection, to make it accessible to the replication machinery [37,38,39,40]. Furthermore, through interactions mediated by its C terminus, the N protein interacts with the viral envelope protein M, which is also involved in genome condensation and packaging in the viral particle [41,42,43,44].

**Figure 2:**
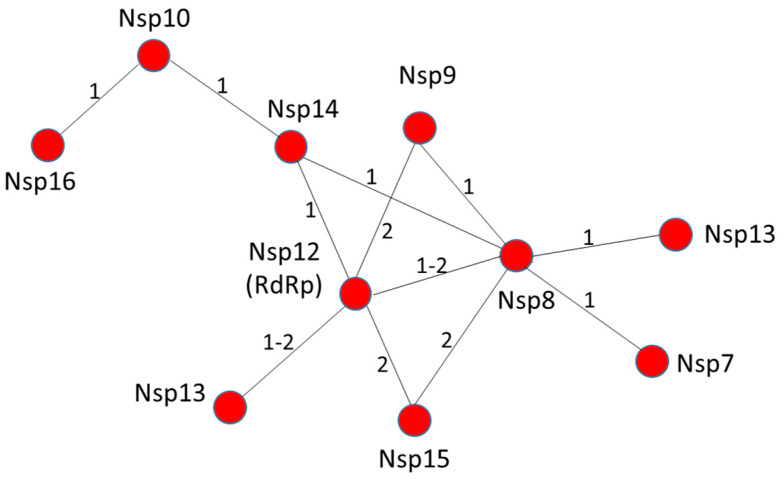
Nsp12 and Nsp8 are a hub for interactions among actors of the RNA replication machinery. Predicted pattern of interactions based on the available literature data. The numbers 1 and 2 on the strings refer to SARS-CoV and MHV, respectively.

Data in the literature on possible interactions among the individual actors of the RNA polymerase machinery of SARS-CoV-2 are still limited. However, based on data for highly homologous proteins from either SARS-CoV (sequence identity >88%) or mouse hepatitis virus (MHV) (sequence identity >40%, Table S1), we reconstituted the likely pattern of interactions among the Nsp proteins constituting the RNA replication/transcription machinery (Figure 2). Among these, we predict that Nsp12 and Nsp8 play a central role in the assembly of the entire RNA polymerase replicative machinery. Nsp12, a RNA-dependent RNA polymerase is the key enzyme mediating the synthesis of all viral RNA molecules [64]. Biochemical studies have proved that Nsp12 from SARS-CoV exhibits low processive RNA synthesis in vitro, as it requires the presence of Nsp7 and Nsp8 to bind nucleic acid and perform efficient RNA synthesis [28,29]. The direct association between Nsp8 and Nsp12 has been reported in several coronaviruses and it is a feature that is likely shared by most, if not all, coronaviruses [65,66]. We also predict that Nsp12 of SARS-CoV-2 is able to interact with Nsp13 helicase based on studies of the highly homologous Nsp12 and Nsp13 from SARS-CoV (96.4 and 99.8% sequence identity, respectively). Indeed, Nsp12 of SARS-CoV can enhance the helicase activity of Nsp13 through a direct protein–protein interaction [59]. The positive regulation of Nsp13 by Nsp12 is an important event in viral replication. Indeed, mutation of specific conserved residues of Nsp13 can either negatively impact or block replication of the arterivirus, equine arteritis virus (EAV) [67,68]. Finally, similar to SARS-CoV, Nsp12 is most likely able to associate with Nsp14 [29], Nsp5 and Nsp9 [69] (Figure 2).

Nsp8 and Nsp7/Nsp8 complex can also bind and enhance the endoribonuclease NendoU activity of MERS-CoV Nsp15 in vitro [70]. This result agrees with previous evidence showing that Nsp15 from MHV colocalizes and interacts in vivo with Nsp8 and Nsp12 [71]. However, whether Nsp15 belongs to the RNA replication machinery is still an open question. Finally, Nsp8 deletion or disruption of the protease cleavage site between Nsp7 to Nsp9, which is necessary to correctly process the corresponding proteins, both result in impaired RNA synthesis and a lethal phenotype in MHV [72]. Altogether, these findings identify the Nsp12-Nsp8 complex as a key hub for the viral replication machinery (Figure 2).

### 3.2. RNA Polymerization Requires Nsp12 and Cofactors Nsp7, Nsp8

**Figure 3:**
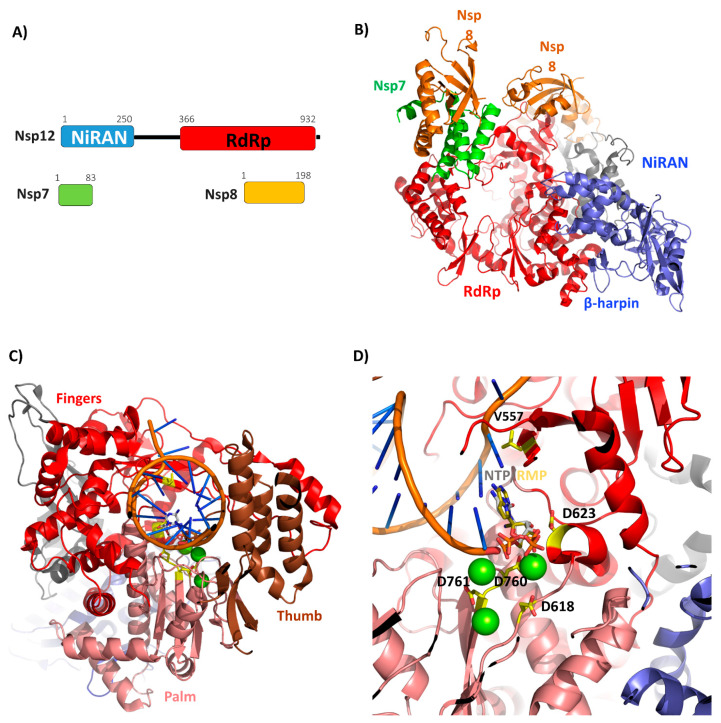
SARS-CoV-2 Nsp12-Nsp7-8 complex. (**A**) Domain organization of Nsp12, with its Nsp7 and Nsp8 cofactors, according to Pfam. (**B**) Cartoon representation of Nsp12 SARS-CoV-2 bound to Nsp7 and Nsp8 cofactors (PDB code 7BTF). (**C**) Model of SARS-CoV-2 elongation complex. The positions of the RNA template/primer and of the divalent cations were obtained from the structural alignment of the complex in panel A with the elongation complex from SARS-CoV-2 (PDB code 7BV2), while the position of NTP was obtained from the alignment with the polymerase of norovirus (PDB code 3H5Y). The three subdomains of the polymerase domain, finger (residues 366–581 and 621–679), palm (residues 582–620 and 680–815), and the thumb (residues 816–920) are shown in red, salmon and brown, respectively. (**D**) A zoom of the catalytic site showing the position of the incoming NTP, Remdesivir monophosphate (RMP) (in stick) and divalent cations (as green spheres). The conserved Asp residues that play a key role in the NTP and Mg^2+^ binding and Val557 (involved in Remdesivir resistance) are shown as yellow sticks.

SARS-CoV-2 RNA polymerization relies on the main polymerase, Nsp12, also denoted as RNA-dependent RNA polymerase, RdRp. Nsp12 is a large enzyme (932 residues) characterized by two conserved domains: the NiRAN and the polymerase domains (Figure 3A). The structure of SARS-CoV Nsp12 bound to the Nsp7 and Nsp8 cofactors has recently been determined using cryo-EM [48]. During the preparation of this manuscript, the structure of SARS-CoV-2 Nsp12 has also been reported [57]. Consistent with the high sequence identity between the Nsp12 form SARS-CoV and SARS-CoV-2 (94%), the two structures are nearly identical, as indicated by their root mean square deviation of 0.8 Å for 1078 Cα atoms [57]. In both structures, Nsp12 is complexed with the two co-factors Nsp7 and Nsp8 (Figure 3B). The observed interactions in the complex structure Nsp12-Nsp8-Nsp7 are compatible with a previous work addressing the impact of residues of Nsp7 and Nsp8 on their interactions with Nsp12 [29].

The N-terminal domain of Nsp12, which has been shown to be essential for viral growth in both equine arteritis virus (EAV) and SARS-CoV [73], is conserved in all nidoviruses endowed with nucleotidylation activity. Therefore, it is named NiRAN from nidovirus RdRp-associated nucleotidyltransferase (Figure 3A,B) [73]. The structure of the NiRAN domain was only partially described in the structure of SARS-CoV Nsp12, whereas it is fully complete in the last released structure of the SARS-CoV-2 Nsp12-Nsp7-Nsp8 complex [57]. Overall, the form of this domain is characterized by an α + β fold composed of eight α helices and a five stranded β-sheet (Figure 3B). In addition, an N-terminal β-hairpin (residues 29–50) interacts with the palm subdomain of the RdRp domain (Figure 3B) [57]. This information provides structural tools to understand the functional role of the NiRAN domain in Nsp12. Indeed, although the exact role of this domain is not fully clear, structural similarity analyses using DALI suggest that the NiRAN domain of SARS-CoV-2 Nsp12 displays structural features of kinase-like folds [48]. Indeed, using DALI we identified two kinases as the most similar structures, the serine/threonine kinase PRP4 homolog (PDB code 6PJJ, DALI Z = 10.5, RMSD = 2.8 Å, seqid 15%) and tyrosine-protein kinase JAK1 (PDB code 6C7Y, DALI Z = 10, RMSD = 2.8 Å, seqid 11%) [74]. Given the importance of the NiRAN domain in viral growth [73], this structural observation suggests a further tool for drug development using kinase inhibitors.

The RNA polymerase C-terminal domain of Nsp12 (residues 366–920) from SARS-CoV-2 adopts a conformation that has been described as a cupped right hand, constituted of finger, palm and thumb subdomains [48,57] (Figure 3C). Biochemical and structural studies of polymerases from other viruses, e.g., poliovirus and foot-and-mouth disease virus, have defined the catalytic cycle of the RNA polymerase as a multi-step process composed of successive steps [75,76]. Catalytic residues can be identified in SARS-CoV-2, upon alignment with poliovirus RNA polymerase, whose catalytic residues are known, with the two aspartic acids Asp618 and Asp760 (Supplementary Materials, Figure S1). Consistently, the D760A mutant of SARS-CoV Nsp12 is unable to synthetize RNA [29]. Together with these two aspartic acids, Asp623 and Asp761 are also involved in the recognition of the NTP triphosphate and divalent cations, respectively [48,57]. The recent crystal structure of SARS-CoV-2 Nsp12-Nsp7-Nsp8 has confirmed that both Asp760 and Asp761 are involved in the coordination of the two important magnesium ions at the catalytic center (Figure 3D). Importantly, a key initiation step is the addition of the first one or two nucleoside triphosphates (NTP) onto a primer, to form a stable and processive elongation complex [75,76]. The structure of the elongation complex of SARS-CoV-2 contains 14 bases in the template strand and 11 bases in the primer strand. This double-stranded RNA helix contacts all of the three Nsp12 subdomains (finger, palm, thumb, Figure 3C). Most of protein-RNA interactions are mediated with the RNA phosphate-ribose backbones, with many interactions directly to 2′-OH groups, thus providing a basis to distinguish RNA from DNA [58]. Notably, the position of the RNA primer is nearly superimposable to that obtained for the well characterized poliovirus [75,77] (Figure 3D, Figure S1). Also, residues involved in RNA binding and composing the catalytic active site are highly conserved (Figure 3, Figure S1), thus suggesting a similar mechanism of RNA replication. It is interesting to note that apo and complexed Nsp12 are almost identical, with an RMSD of 0.5 Å, thus suggesting that SARS-CoV-2 Nsp12 does not require a conformational switch upon ligand binding. This hypothesis agrees well with the high processivity of viral RNA polymerases [76] since no extra energy is required to switch the enzyme conformation towards activation.

**Figure 4:**
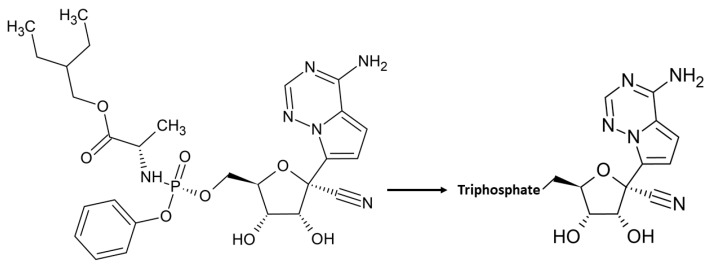
Chemical structure of Remdesivir (GS-5734) and its pharmacologically active nucleoside triphosphate NTP.

Although sequence identity of Nsp12 with RdRP from the Ebola virus (EBOV) is quite poor (16%), structural analysis indicates the conservation of the polymerase active site. Remdesivir, the nucleotide inhibitor of the EBOV RdRP has been recognized as a promising antiviral drug against a wide array of RNA viruses including filoviruses, arenaviruses, paramyxoviruses, and other coronaviruses with divergent RdRp, such as SARS-CoV, MERS-CoV, bat CoV, and the new SARS-CoV-2 strains [78,79,80,81,82,83] in cultured cells, mice and nonhuman primate models [78,84,85]. Remdesivir is a prodrug, which is metabolized into its active form (GS-441524), which causes a decrease in viral RNA production [78] (Figure 4). The compound has a 1′-cyano group, which provides potency and selectivity toward viral RNA polymerases, and a monophosphate promoiety to enhance intracellular metabolism into the active triphosphate metabolite [86] (Figure 4). Remdesivir triphosphate is able to inhibit EBOV replication with half maximal effective concentrations (EC50) in the sub-micromolar range [78] by blocking viral RNA synthesis [78]. The mechanism of inhibition is a delayed chain termination of nascent viral RNA, as described for several viral RdRP, including EBOV, MERS, Nipah (NIV) and respiratory syncytial virus RSV [78,87,88,89]. In all cases, Remdesivir triphosphate inhibits transcription by competing with the incorporation of natural NTP counterparts [87,89]. This finding has been recently corroborated for SARS-CoV-2 by the determination of the structure of Nsp12-Nsp7-Nsp8 in complex with RNA template/primer and Remdesivir [58] (Table 1). This structure has shown that Remdesivir monophosphate (RMP) is covalently incorporated at the 3′ end of the primer strand [58]. As shown in Figure 3D, this position fully overlaps with the +1 position of a natural NTP. Additionally, it was previously shown for MHV and SARS-CoV [79] that a mutation of a conserved valine residue of Nsp12, corresponding to V557L in SARS-CoV-2, confers low-level resistance to Remdesivir; it impairs fitness and attenuates virulence. This mutation is located in proximity to the NTP binding site of Nsp12 [58] (Figure 3D).

Significantly, a recent study revealed that Remdesivir is effective in the control of SARS-CoV-2 in vitro [81]. From the clinical point of view, Remdesivir is being used in a small number of SARS-CoV-2 positive patients for “compassionate use” based on the patients’ worsening clinical status. One of the first cases in the USA is responding well to the nucleoside analogue with an immediate improvement after intravenous injection [90]. Nevertheless, it is not yet approved for general use and clinical trials are currently underway to determine its safety and efficacy [91]. Structurally-based improvements of this drug may constitute a valid tool to enhance its specificity and efficiency against coronaviruses.

### 3.3. RNA Proofreading and mRNA Capping through the Bifunctional Protein Nsp14 and Cofactor Nsp10

**Figure 5:**
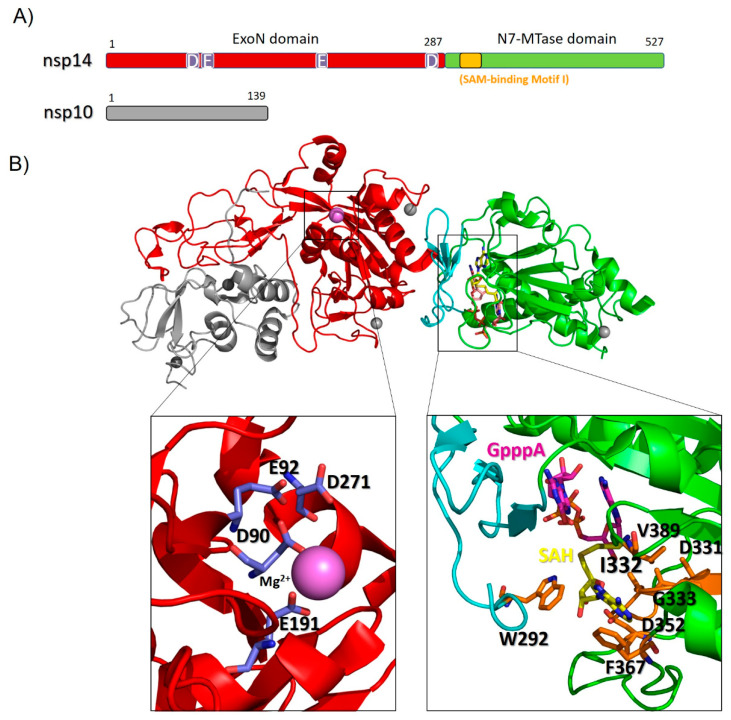
Homology model of SARS-CoV-2 Nsp14-Nsp10 complex. (**A**) Domain organization of Nsp14 and Nsp10; (**B**) Cartoon representation of the homology model of the complex, computed with MODELLER using the structure of its homolog from SARS-CoV as a template (PDB code 5C8S, covered region 1−131 in Nsp10 and 1−525 in Nsp14). Zinc atoms are shown as grey and a Mg^2+^ ion as magenta spheres. Zooms of the catalytic sites are shown in the insets. Catalytic residues of the ExoN domain (left inset) are shown as blue sticks, those of the N7-MTase domain (right inset) are shown as orange sticks; the cap-precursor GpppA (pink), a SAH (demethylated form of SAM) ligand (yellow) and the SAM-binding motif residues (orange) are also represented as sticks.

Nsp14 of SARS-CoV-2 contains two domains with different functions, as identified by the PFAM database (Figure 5A). The N terminal domain (ExoN) is endowed with exoribonuclease activity and includes three conserved motifs: motif I (DE), II (E) and III (D). Due to this feature, Nsp14 is included as a “DEED outlier” into the superfamily of DEDD exonucleases [55,92], which embrace enzymes with proofreading activity [92,93]. In line with this observation, ExoN knockout mutants of SARS-CoV and murine hepatitis virus (MHV) were shown to accumulate a high number of mutations [94,95]. The carboxy-terminal part of Nsp14, containing (N7 guanine)-methyl transferase activity, is involved in the viral mRNA cap synthesis. The RNA final cap has several important biological roles in viruses as it is critical for the stability of mRNAs, for their translation and to evade the host immune response. Indeed, uncapped RNA molecules are degraded in cytoplasmic granular compartments and may be detected as “non-self” by the host, therefore triggering innate immune responses [96,97].

A homology model of the complex between Nsp14 and Nsp10 of SARS-CoV-2 is reported in Figure 5B, based on the structure of homologous proteins of SARS-CoV [54,55] (Table S1). In this complex, the co-factor Nsp10, composed of a helical domain and an irregular β-sheet region followed by a loop region at its C-terminus, forms multiple interactions with the ExoN domain of Nsp14, likely stabilizing it. Consistent with this, SAXS experiments of Nsp14 from SARS-CoV in the absence of Nsp10 show large conformational changes in the N terminus of Nsp14, which affect the overall shape of the exonuclease fold [54]. Importantly, the interaction with Nsp10 strongly affects the nucleolytic activity of SARS-CoV Nsp14, which is enhanced up to 35-fold [98].

The ExoN domain of Nsp14 presents an α/β fold as do the other members of the DEDD exonuclease superfamily [99]. It is composed of a central twisted β-sheet formed by five β -strands. These, are flanked by α-helices, with the exception of the strand β3 (Figure 5B) [54,55]. Based on its structural alignment with SARS-CoV, the catalytic residues of the ExoN domain of SARS-CoV-2 Nsp14 include the DEED residues Asp90, Glu92, Glu191, Asp272 (Figure 5). A structural alignment using DALI shows that this domain is structurally similar to *E. coli* RNase T and to RNase AS from *M. tuberculosis*, two exonucleases involved in RNA maturation through 5′ processing [100,101]. Similar to these exonucleases, alanine substitution of the four catalytic residues of ExoN, which coordinate a Mg^2+^ ion (Figure 5B), results in a significant reduction of the viral RNA synthesis [94,100,101,102,103]. Altogether, these data suggest that like Nsp12, Nsp14 has a crucial role in SARS-CoV-2 replication through its ExoN domain, as it is involved in maintaining the integrity of the SARS-CoV-2 RNA genome, preventing and repairing mutations [95,104]. In this context, susceptibility of EBOV to Remdesivir also involves the proofreading exoribonuclease, Nsp14 [79]. For EBOV RNA polymerase, the incorporation of the nucleotide analogue at position n causes inhibition of RNA synthesis predominantly at position n + 5 [88,89] whereas in the case of MERS, arrest of RNA synthesis occurs at position n + 3, with these three nucleotides likely protecting the inhibitor from excision by the viral 3′–5′ exonuclease activity [89]. In both cases, delayed chain-termination could be due to inhibitor-induced structural changes of the newly synthesized double stranded RNA [105,106]. Importantly, a mutant lacking ExoN was significantly more sensitive to Remdesivir, suggesting that this nucleoside analogue, once incorporated into viral RNA, can be removed by the exoribonuclease activity of Nsp14 during proofreading [79,107]. Therefore, nucleoside analogues that effectively inhibit viral RNA replication must either evade detection by the exonuclease or outcompete exonuclease activity [108]. In this case, chemical modifications of the nucleoside analogues to skip recognition by Nsp14, or simultaneous inhibition of Nsp12 and Nsp14, would provide a synergistic action in the inhibition of RNA synthesis and be a powerful strategy against SARS-CoV-2.

A flexible hinge region consisting of a loop and three strands (shown as cyan in Figure 5B) separates the ExoN domain from the N7-MTase domain of Nsp14 and is highly conserved across CoVs. This region allows lateral and rotational movements of the two domains to coordinate the two different enzymatic activities of Nsp14 (Figure 5B) [92]. Following this hinge region, the N7-MTase domain is an (N7 guanine)-methyl transferase involved in RNA capping, which operates by demethylating its co-enzyme S-adenosyl methionine (SAM). This domain shows unique structural features as it displays an atypical fold, different from the canonical Rossmann fold of the virus RNA MTase [54,109]. In addition, it does not belong to any of the classes of SAM-dependent MTases [110,111,112]. Indeed, a typical Rossmann fold embeds a central core of β-sheets composed of seven parallel β-strands with at least three a-helices on each side [109]. Differently, the β sheet of the N7-MTase domain of Nsp14 is formed by five β -strands instead of seven (Figure 5B). SARS-CoV Nsp14-Nsp10 crystal structure [55], together with alanine scanning mutagenesis [113] and cross-linking experiments, revealed two clusters of residues that are key for the N7-MTase activity (Figure 5B) [113,114,115]. The first cluster is a canonical SAM-binding motif I (DxGxPxG/A) and includes Asp331, Gly333, Pro335 and Ala337 (Figure 5B), where SAM is the methyl donor in the (N7 guanine)-methyl transferase reaction catalyzed by Nsp14. A second cluster forms a pocket that holds the GTP of the mRNA cap structure in close proximity of the methyl donor SAM (Figure 5B). The binding mode of the functional ligands, the cap-precursor guanosine-P3-adenosine-5′,5′-triphosphate (GpppA) and the product of SAM demethylation, S-adenosyl Homocysteine (SAH), occurs with no significant structural changes in the enzyme [55]. These studies have helped to shed light on the mechanism of RNA cap formation, which also involves several other protein actors, as detailed below.

### 3.4. SARS-CoV-2 Capping Machinery Involves Nsp13, Nsp14, Nsp16 and the co-Factor Nsp10

#### 3.4.1. RNA Cap Synthesis in Coronaviruses

**Figure 6:**
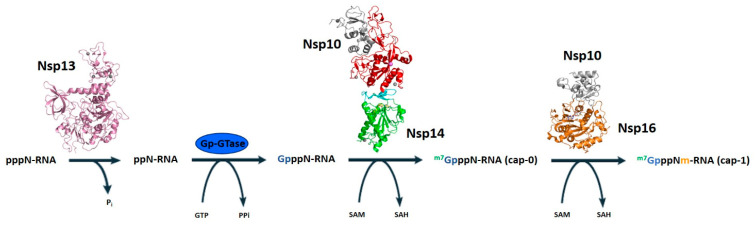
The mRNA cap synthesis process in SARS-CoV-2. The process is performed by the sequential action of four enzymes: Nsp13 (pink), a still unknown GTase, Nsp14 (red) and Nsp16 (orange). The presence of the co-factor Nsp10 (grey) is fundamental for the activity of the last two enzymes.

mRNAs of coronaviruses are protected at their 5′ ends by a cap structure consisting of an N7-methylated GTP molecule linked to the first transcribed nucleotide by a 5′–5′ triphosphate bond. Given the importance of RNA capping for mRNA stability and as a mechanism to evade the host immune response, RNA-capping machineries are an attractive target for antiviral-drug design. In coronaviruses, apart from the N7-MTase domain of Nsp14 described above, the cap synthesis involves several enzymes and the co-factor Nsp10 (Figure 6). The mRNA cap (^m7^GpppN-RNA) is composed of a 7-methylguanosine (^m7^G) linked to the 5′-nucleoside (N) of the RNA chain through a triphosphate bridge (ppp). The process begins with the hydrolysis of the 5′γ-phosphate of the nascent RNA chains (pppN-RNA) by an RNA 5′-triphosphatase, the Nsp13 helicase [21]. Subsequently, a still unidentified GTase transfers a GMP molecule to the 5′-diphosphate of the RNA chains (ppN-RNA), leading to the formation of GpppN-RNA. Then, the cap structure is methylated at the N7 position of the guanosine by the C-terminal N7-MTase domain of Nsp14, forming cap-0 (^m7^GpppN-RNA), using SAM as a methyl donor. Finally, Nsp16 (SAM)-dependent 2′-o-methyltransferase activity promotes the addition of a methyl group on the ribose 2′-O position of the first transcribed nucleotide to form cap-1 (^m7^GpppNm-RNA) [52,53] (Figure 6). In the last steps, the cofactor Nsp10 acts as an allosteric activator [52,53].

#### 3.4.2. Start of mRNA Capping by the Multi-Functional Nsp13 Helicase

Helicases are versatile NTP-dependent enzymes that are widespread in all kingdoms of life including (+) RNA viruses with genome greater than 7 kb [116]. They are classified into six superfamilies (SF1 to SF6) and are known to be critically involved in several processes connected to nucleic acid metabolism [116]. Helicases are required for the unwinding of dsDNA and/or dsRNA substrates, for displacing proteins bound to nucleic acid or remodeling DNA or RNA secondary structures and for translocating along double-strand nucleic acid without unwinding [68]. Sequence conservation analysis shows that Nsp13 of SARS-CoV-2 belongs to the SF1 superfamily and shares many structural features with the eukaryotic Upf1 helicase, a key factor in nonsense-mediated mRNA decay in cells [117]. Like other coronaviruses, Nsp13 exhibits multiple enzymatic activities, which include not only the hydrolysis of NTPs required in the capping mechanism (Figure 6), but also unwinding of RNA duplexes with 5′–3′ directionality and the RNA 5′-triphosphatase activity [118,119]. Additionally, RNA unwinding activity is stimulated by the interaction with the RdRP Nsp12 [120]. Nsp13 is highly conserved in all coronaviruses and is a key enzyme in viral replication [121,122], two observations which make it a promising target for antiviral therapies [68]. In this context, a potent non-competitive inhibitor (SSYA10-001) blocks viral replication by inhibiting the unwinding activity of the helicase Nsp13 [123], not only in SARS-CoV but also for two other coronaviruses, MHV and MERS-CoV [124].

**Figure 7:**
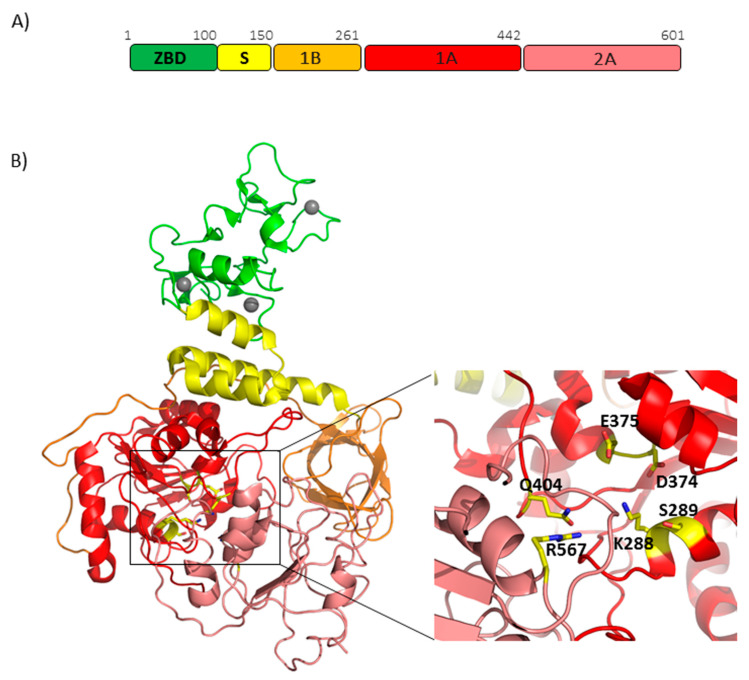
SARS-CoV-2 Nsp13 helicase. (**A**) Domain organization of SARS-CoV-2 Nsp13. (**B**) Cartoon representation of the homology model of SARS-CoV-2 Nsp13, obtained using MODELLER based on the crystallographic structure of the SARS-CoV (PDB code 6JYT, covered region 1-596). The colors of the protein domains are indicated in panel A (ZBD-green, stalk-yellow, 1B-orange, 1A-red and 2A-salmon). Three zinc atoms are shown as grey spheres. In the inset, the key conserved residues responsible for NTP hydrolysis are drawn as sticks.

Nsp13 of SARS-CoV-2 shares the same structure as that of SARS-CoV [59], given the extremely high conservation of protein sequences (Table S1), and consists of five domains which fold in a triangular pyramid shape (Figure 7B). A similar organization was observed for MERS helicases [60]. Three domains named 1A and 2A and the 1B domain are arranged to form the triangular base, leaving the remaining two domains, the N-terminal Zinc binding domain (ZBD) and the stalk domain, at the apex of the pyramid (Figure 7B). Mutagenesis and structural alignments demonstrated that the 1B, 1A and 2A domains are responsible for NTP activity and nucleic acid binding, whereas other functional information about the structural coordination of these five domains in helicase activity have been deduced through the H/D exchange assays. As explained above, the activity of Nsp13 is enhanced by Nsp12 through direct interaction, with the interaction region on Nsp13 mapped on the ZBD domain and the 1A domain [59]. Given the high sequence conservation of these two proteins, their association can be considered a common feature across CoV [59].

#### 3.4.3. End of mRNA Capping by 2′-o-Methyl Transferase Nsp16 and Nsp10, an Allosteric Activator and a Molecular Connector?

**Figure 8:**
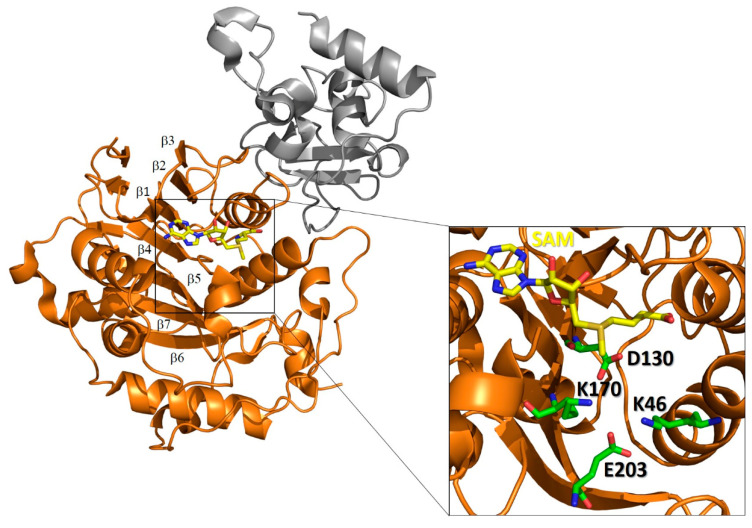
Structure of the SARS-CoV-2 Nsp10-Nsp16 complex. Cartoon representation of the crystal structure complex (PDB code 6W4H) of Nsp16 (orange) in complex with Nsp10 (grey). The inset shows the catalytic site of Nsp16. Catalytic residues (green) and the SAM ligand (yellow) are shown in stick form.

Several X-ray structures of Nsp16 are deposited in the PDB, including those from SARS-CoV, MERS-CoV and more recently of SARS-CoV-2 (Table 1). In all of these structures, Nsp16 is complexed with the cofactor Nsp10 and presents a similar topological organization [53,125]. In particular, Nsp16 possesses the typical fold of the class I MTase family, comprising a seven-stranded β sheet flanked by α helices with the characteristic reversed β hairpin at the carboxyl end of the sheet (β6−β7) (Figure 8) [112]. The catalytic site cleft of Nsp16 contains the conserved K-D-K-E catalytic tetrad, which is peculiar to SAM-dependent 2′-o-methyltranferases. Specifically, the four residues Lys46, Asp130, Lys170 and Glu203 are predicted to be catalytic (Figure 8) [112]. In addition, a conserved SAM-binding pocket is located at the C-terminal end of strands β1 and β2, as in the case of other SAM-dependent MTases [126] (Figure 8). Two zinc ions were identified as bound to two zinc finger regions and found to be indispensable for the binding of the RNA chains in a nonselective manner (Figure 8) [56,127,128].

Mutagenesis studies confirmed the central role played by the K-D-K-E tetrad of Nsp16 for the 2 O-MTase activity [52]. Infection of small animal models with viruses expressing Nsp16 mutants showed a decrease in viral titers. These findings suggest that the inhibition of the viral 2 O-MTase activity, and the consequent production of an incompletely capped RNA, might stimulate the detection of viral RNA by pathogen-associated molecular patterns (PAMPs) and induce a host antiviral response. Indeed, it was shown that the incompletely capped RNA can be detected by the cytosolic RIG-I-like receptors (RLRs) RIG-I or Mda5 [125,129,130,131], which stimulate the production of type I interferon (IFN). The resulting IFN secretion induces the host antiviral response mediated by IFN-induced protein with tetratricopeptide repeats (IFIT) proteins [132,133].

As shown in the case of SARS-CoV, Nsp16 needs to interact with Nsp10 to become active [53]. Indeed, SARS CoV Nsp16 alone has low affinity for both m7GpppA-RNA and m7GpppA cap analogue, and the interaction between the two proteins increases RNA-binding affinity [53]. Similar results were observed for MERS CoV Nsp16/Nsp10 complex [125].

**Figure 9:**
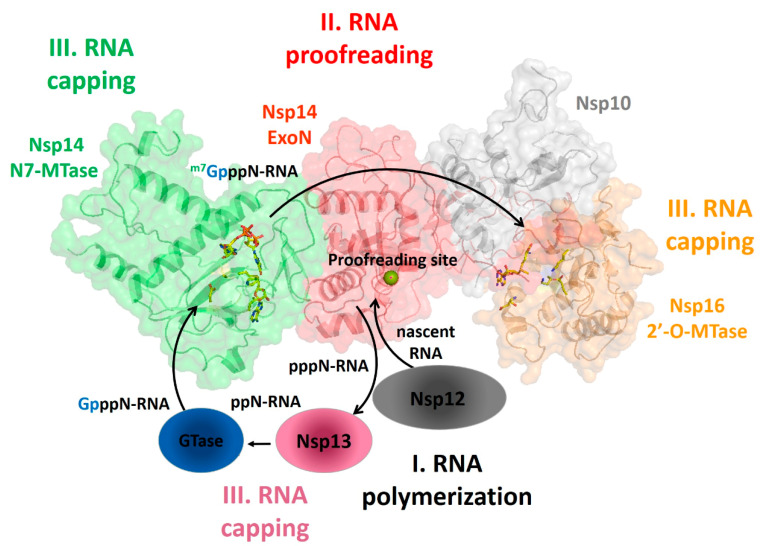
Nsp10 as a molecular connector between proofreading and capping activities. Cartoon and surface representation of the macromolecular complex generated upon superposition of Nsp10 in the two complexes Nsp14-Nsp10 and Nsp16-Nsp10. This model was obtained upon superposition of Nsp10 cofactors of the Nsp10/Nsp14 and Nsp10/Nsp16 complexes, using CCP4. A nascent RNA strand polymerized by Nsp12 (grey oval) is proofread by Nsp14, dephosphorylated by Nsp13 and then capped by GTase, Nsp14 and finally Nsp16.

As previously discussed, the cofactor Nsp10 plays a similar activating role for Nsp14, as demonstrated for SARS-CoV [98]. Therefore, we explored the possibility of the formation of a ternary complex Nsp10/Nsp14/Nsp16 by verifying the compatibility of binding of Nsp14 and Nsp16 to Nsp10 (see caption for Figure 9 for details). As shown in Figure 9, interacting regions on Nsp10 with Nsp14 and Nsp16 are perfectly complementary, thus allowing for a simultaneous interaction of Nsp10 with both Nsp14 and Nsp16. This finding suggests it is likely that Nsp10 works not only as an allosteric activator of the two enzymes but also as a molecular connector, joining three catalytic sites together in space (Figure 9). As shown in Figure 9, a ternary complex Nsp10-Nsp14-Nsp16 would keep the proofreading site of Nsp14 close in space to the two catalytic sites responsible for capping on Nsp14 and on Nsp16. This ternary complex is likely only a part of a large RNA polymerase complex, which is further hold together by the known interactions between the ExoN domain of Nsp14 and Nsp12 [29] and between Nsp13 and Nsp12 [59], although fine details of these interactions are still unknown (Figure 9). In the case of a nucleotide mismatch, the nascent RNA strand can be efficiently moved from Nsp12 to the ExoN site of the RNA polymerase complex for excision (Figure 9). Final capping is then provided by the sequential activities of close-in-space enzymes. Namely, the Nsp13 helicase, the GTase and then the N7-MTase activity of Nsp14 and the 2′-OMTase activity of Nsp16 (Figure 9), thus completing the nascent RNA strand with the cap1 structure at its 5′ end.

## 4. Concluding Remarks

Over the past ten years, we have observed the emergence of many different coronaviruses, that have caused serious diseases like SARS in 2002, MERS in 2012 and currently, COVID19. It is more than likely that coronaviruses will emerge again in the near future due to their ability to mutate, recombine and infect different hosts, as we have just observed for SARS-CoV-2, which has evolved from a bat disease to a human disease outbreak. A possible strategy is to identify those processes that are more preserved in all coronaviruses and deeply understand their mechanisms. By inhibiting these processes there is a great chance of developing a pancoronaviral therapeutic strategy. As discussed in this review, molecular actors responsible for RNA replication are among the most conserved coronaviral proteins and therefore deserve deep understanding of their structural properties. The composition and structural organization of the replicase complex of SARS-CoV-2 has not been thoroughly studied, although research in the field is progressing fast. However, there are many lessons to be learnt from studies of the RNA replication of similar viruses, as reported in this review. These studies, both structural and functional, have identified and described enzymes responsible for RNA polymerization, proofreading and final capping mechanisms to produce stable RNA. However, an important role is also played by cofactors, which mediate protein–protein interactions and thus conduct the orchestra of RNA processing enzymes to improve their efficiencies. Gaining a complete picture of the intricate process of RNA replication of SARS-CoV-2 significantly improves our ability to design therapeutic tools to reduce disease burden.

## Figures and Tables

**Table 1:** Available structural information on putative SARS-CoV-2 RNA replication machinery actors.

Target	Function	PDB Code [Reference]	Source	Seqid (%)
Nsp7	Cofactor of Nsp12	1YSY [45]; 2KYS [46]; 2AHM (in complex with Nsp8) [47]; 6NUR (complex with Nsp8 e Nsp12) [48]	SARS-CoV	98.8
Nsp8	Cofactor of Nsp12	2AHM (complex with Nsp7) [47]; 6NUR (complex with Nsp7 e Nsp12) and 6NUS (complex with Nsp12) [48]	SARS-CoV	97.5
Nsp9	RNA binding protein	6W4B, 6W9Q, 6WC1 [49]; 1QZ8 [50]; 1UW7 [51]	SARS-CoV-2 SARS-CoV	100 97.4
Nsp10	Cofactor of Nsp16 and Nsp14	6W61, 6W75 6W4H (complex with Nsp16); 2XYR, 2XYV, 2XYQ (complex with Nsp16) [52]; 3R24 (complex with Nsp16) [53]; 5NFY (complex with Nsp14) [54]; 5C8S, 5C8T, 5C8U (complex with Nsp14) [55]; 2GA6 [56]	SARS-CoV-2 SARS-CoV	100 98.5
Nsp12	RNA-directed RNA polymerase	6M71 and 7BTF (in complex with Nsp7 and Nsp8 [57];7BV1 (complex with Nsp7 and Nsp8) and 7BV2 (complex with Nsp7 and Nsp8, RNA template/primer and Remdesivir, [58];6NUS (complex with Nsp8) and 6NUR (complex with Nsp7 and Nsp8) [48]	SARS-CoV-2 SARS-CoV	100 96.4
Nsp13	Helicase, 5′ triphosphatase	6JYT [59]; 5WWP [60]	SARS-CoV MERS-CoV	99.8 72.2
Nsp14	3′–5′ exoribonuclease, ExoN; Guanine-N7 methyltransferase, N7 MTase	5C8S, 5C8U, 5C8T (complex with Nsp10) [55]; 5NFY (complex with Nsp10) [54]	SARS-CoV	95.1 94.9
Nsp15	NendoU, Uridylate-specific endoribonuclease	6W01, 6VWW [61]; 2H85 [62]; 2RHB [63]	SARS-CoV-2 SARS-CoV	100 88.0
Nsp16	2’-O-ribose methyltransferase	6W4H, 6W75, 6W61 (complex with Nsp10); 2XYR, 2XYV, 2XYQ (complex with Nsp10) [52]; 3R24 (complex with Nsp10) [53]; 5YN5 (complex with Nsp10).	SARS-CoV-2 SARS-CoV MERS-CoV	100 93.5 66.1

## Supplementary Materials

The following are available online at https://www.mdpi.com/2073-4409/9/5/1267/s1, Figure S1: Model of SARS-CoV-2 elongation complex, Table S1: Sequence identities of SARS-CoV2 Nsps with homologous proteins.

## Abbreviations

WHO	World Health Organization
DMV	Double-membrane vesicles
PDB	Protein Data Bank
SARS	Severe acute respiratory syndrome
MERS	Middle East respiratory syndrome
EBOV	Ebola virus
NIV	Nipah virus
RSV	Respiratory syncytial virus
EAV	Equine arteritis virus
RNP	Ribonucleocapsid
Nsp	Non-structural protein
RdRP	RNA dependent RNA polymerase
NiRAN	Nidovirus RdRp-associated nucleotidyltransferase
NTP	Nucleoside triphosphate
PAMP	Pathogen-associated molecular pattern
