# Supplementary material for: A Structural View of SARS-CoV-2 RNA Replication Machinery: RNA Synthesis, Proofreading and Final Capping
Source: Cells. 2020 May 20;9(5):1267. doi: 10.3390/cells9051267 (PMC7291026; doi:10.3390/cells9051267)
Supplement: Supplementary file 1 [file cells-09-01267-s001.pdf]

## Supplementary Material

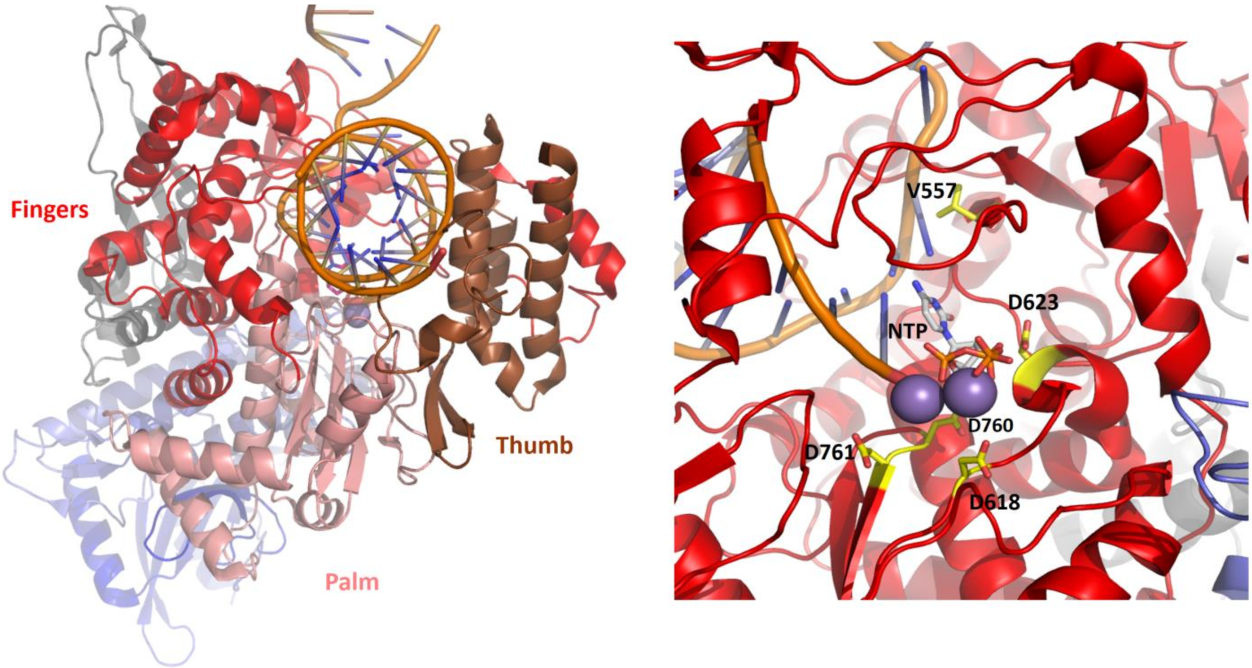


**Figure S1.** Model of SARS-CoV-2 elongation complex; The positions of the RNA template and primer were obtained from the structural alignment of SARS-CoV-2 Nsp12 with the elongation complex of poliovirus (PDB code 3OL6). The position of $Mg^{2+}$ ions was taken from the structure of norovirus (PDB code 3H5Y). The three subdomains of the polymerase domain, fingers (residues 366-581 and 621-679), palm (residues 582-620 and 680-815), and the thumb (residues 816-920) are shown in red, salmon and brown, respectively. Right) A zoom of the catalytic site showing the position of the incoming NTP and divalent cations (grey). The conserved Asp residues playing a key role in the NTP and divalent cations binding and Val557 (involved in Remdesivir resistance) are shown as yellow sticks.

**Table S1.** Sequence identities of SARS-CoV2 Nsps with homologous proteins. Identities are reported with homologs from SARS-CoV and MHV. The binary alignments were performed using Lalign Server tool.

| **SARS-CoV-2** | SARS-CoV | MHV |
|---|---|---|
| Nsp7 | 98.8 | 43.5 |
| Nsp8 | 97.5 | 45.3 |
| Nsp9 | 97.3 | 46.9 |
| Nsp10 | 97.1 | 52.5 |
| Nsp12 | 96.4 | 66.3 |
| Nsp13 | 99.8 | 67.3 |
| Nsp14 | 95.1 | 82.5 |
| Nsp15 | 88.7 | 44.9 |
| Nsp16 | 93.3 | 63.9 |
